# Impact of pregnancy on papillary thyroid carcinoma prognosis

**DOI:** 10.11604/pamj.2021.38.261.22762

**Published:** 2021-03-15

**Authors:** Yasmine Driouich, Nassim Essabah Haraj, Siham El Aziz, Asma Chadli

**Affiliations:** 1 Endocrinology, Diabetology and Metabolic Disease Department, Ibn Rochd University Hospital of Casablanca, Casablanca, Morocco,; 2 Neurosciences and Mental Health Laboratory, Faculty of Medicine and Pharmacy, University Hassan II Casablanca, Casablanca, Morocco

**Keywords:** Papillary carcinoma, thyroid, pregnancy, remission, recurrence, prognostic

## Abstract

**Introduction:**

thyroid carcinoma is more frequent in women of reproductive age. It can affect both fertility and the course of pregnancy. The aim of the study was to investigate the influence of pregnancy as a factor of recurrence or progression, on the prognosis of thyroid carcinoma.

**Methods:**

we conducted a retrospective cohort study of 117 young female patients followed up for papillary thyroid carcinoma (PTC) at the Department of Endocrinology, Diabetology and Metabolic Diseases of Ibn Rochd University Hospital of Casablanca, from January 2010 to December 2018, divided into 2 groups: group 1 composed of pregnant patients (n=42) and group 2 being the control group (n=75). Statistical analysis was made using SPSS software version 22.0.0.

**Results:**

average age of pregnant patients was 35 ± 6.5 years old. Mean duration between first pregnancy and treatment completion was 4.4 ± 3.1 years. Over an average treatment duration of 14.4 months in postpartum, 30 patients were in remission (thyroglobulin (Tg) <1μg/l, negative thyroglobulin antibody and no morphological abnormality), while 12 had persistent symptoms (detectable Tg/thyroglobulin antibody and/or morphological abnormality). Thyroid stimulating hormone (TSH) during pregnancy was on average 0.83 mIU/l. Cancer progression was correlated with persistence of thyroid cancer to treatment before pregnancy (p = 0.01), pre-existing distant or locoregional metastases (p = 0.02) and delayed administration of radio-iodine therapy (p = 0.01). Interval between diagnosis and pregnancy, TSH level during pregnancy or the pre-conception thyroglobulin level did not have a statistically significant impact. Pregnancy was not associated with progression or recurrence of thyroid cancer (adjusted risk ratio 1.04, 95% confidence interval 0.91-1.32).

**Conclusion:**

this study shows that pregnancy has no impact on recurrence or progression of thyroid cancer in patients declared in remission prior to conception.

## Introduction

The incidence of thyroid cancer is increasing worldwide [1]. Papillary thyroid carcinoma (PTC) is most common histological type, accounting for 80% of cases. This increase affects all ethnic and age groups with a higher risk in women under 45 years [2]. It is the second most diagnosed cancer during pregnancy and the postpartum period, with a prevalence of 14 per 100,000 live births [1-3]. During a normal pregnancy, the increase of human chorionic gonadotropin (HCG) in the first quarter, and increase of concentration of estrogen, have stimulating effects on the thyroid gland [4]. Since pregnancy stimulates normal and nodular thyroid growth in healthy women, it is reasonable to worry that pregnancy in women with thyroid carcinoma can boost cells growth, even if declared in remission. Multiple authors have studied papillary thyroid carcinoma management and prognosis diagnosed before pregnancy, with conflicting results, and have suggested an association between PTC and reproductive variables such as estrogen and HCG [5,6]. Although there were earlier reports of pregnancy accelerating thyroid cancer growth, more recently many other retrospective series have reported that pregnancy has no significant impact on the outcomes of well-differentiated thyroid cancer [7,8]. However, no consensus is available on the influence of pregnancy on thyroid cancer [3], apart from the accuracy of ideal timing of surgery [9]. Thus, the purpose of the study was to determine the effect of pregnancy in thyroid cancer survivors at risk for progression or recurrence of the disease.

## Methods

We conducted a retrospective cohort study from January 2010 to December 2018, on the patients followed up for papillary thyroid carcinoma. The recruitment site was the Endocrinology Service - CHU Ibn Rochd Diabetology of Casablanca.

**Inclusion criteria:** our study included 117 patients selected according to the following criteria: age <45 years, total thyroidectomy; radioiodine (I-131) therapy indication according to clinical stage; follow-up duration over one year. Our patients were divided into 2 groups: Group 1 (n = 42) experienced pregnancy during postoperative follow-up, group 2 (n = 75) patients who have not been pregnant during follow-up. Thus, patients in group 1 with PTC were followed up for thyroid carcinoma since the pregnancy diagnosis until delivery, with regular postpartum monitoring.

**Variables studied:** parameters studied were: interval between completion of treatment and pregnancy, duration of follow-up, course of pregnancy, obstetrical prognosis, thyroid status in pre/per/postpartum, and postpartum evolution of carcinoma. Each patient was classified according to the World Health Organization classification [10], organized by the 8^th^ edition of the TNM classification (American Joint Committee on Cancer (AJCC)) [11] with a risk stratification as recommended in the American Thyroid Association (ATA) [12]. To determine the progression or recurrence of thyroid cancer during pregnancy, we compared the biological and morphological thyroid assessment before and after pregnancy, from 6 to 24 months after postpartum.

### Definitions

**Remission:** suppressed Tg below 1 ng/ml, or undetectable ultrasensitive Tg (< 0.2 ng/ml), with negative thyroglobulin antibodies (TgAb) and without morphological abnormality on ultrasound.

**Recurrence/progression of the disease were defined by:** detectable Tg levels (>2 ng/ml) or persistent TgAb (with a constant increase and presence of a morphological abnormality on ultrasound).

**Data collection and analysis:** data was collected from medical files, then transferred into an Excel sheet. We used SPSS software, version 22.0 to analyse data. For the descriptive part, the standard deviations, means, and percentages, were calculated to summarize the distributions of qualitative variables in pregnant and non-pregnant women. Pearson Chi 2 test was used to evaluate the association between clinical and obstetrical factors with the progression of thyroid carcinoma; the significance threshold (p) was set at < 0.05. Factors identified as significant by univariate analysis were further investigated using logistic regression.

## Results

Our study included 117 patients with PTC screened from January 2010 to December 2018. The average age in Group 1 (G1) was 35.0 ± 6.5 years, which was statistically higher than the control group (G2), which was 31.2 ± 5.7 years (p = 0.02). All patients underwent thyroidectomy with a levothyroxine substitution after surgery. In only six women (14.2%), TSH levels was less than 1 mIU/L during pregnancy. In 36 women (85.7%), at least one TSH measurement was above 3 mIU/L during pregnancy. Mean TSH level during pregnancy was 0.83 ± 2.15 mIU/L. However, no correlation was found between TSH levels and progression of the disease or thyroglobulin during pregnancy. No significant differences were noted in tumor size nor in extrathyroidal extension (Tumor-node-metastasis (TNM) classification). No deaths were noted. The average time between pregnancy and completion of treatment was 4.4 ± 3.1 years (range 1 to 8) (Table 1).

**Table 1:** general characteristics of patients

		Group 1		Group 2 (control)	
		n = 42	Percentage (%)	n = 75	Percentage (%)
Average Age (years)		35.0 ± 6.5	-	31.2 ± 5.7	-
Average time between completion of treatment and pregnancy (years)		4.4 ± 3.1	-	-	-
Follow-up duration (months)		14.45 ± 2.4	-	18.24±3.7	-
Risk stratification	High	6	15	10	13
	Intermediate	14	33	17	23
	Low	22	52	48	64
Remission rate		30	72	52	70
Carcinoma persistence rate		12	28	23	30

**Course of pregnancy:** the pregnancy was uneventful in all cases except one, which ended in a miscarriage. The most common delivery mode was cesarean section in 59% of cases. The average birth weight was 2865 ± 475 g. Average follow-up duration after the first delivery was 14.45 ± 2.4 months.

**Evolution:** in postpartum, 30 patients were in remission, while 12 patients had persistent cancers, without significant difference between the two groups (p = 1.25) In the 42 women, the average Tg before pregnancy was about 1.75 ng/ml (range: 0 to 12.4 ng/ml) and the average rate after delivery was 1.98ng/ml (range: 0 to 18.5 ng/ml). The difference was not statistically significant (p = 1.00). Persistent cancer was detected in 12 of the 42 pregnant women (28%), one of them showed disease progression after delivery (2.4%) (p = 1.00). Distant metastases were detected in 3 of 42 pregnant patients (7%) and 18 of 75 non-pregnant women (24%) with a significant difference observed between the 2 groups (p = 0.01). Multivariate logistic analysis was performed on the general population (including two groups: group 1 versus the control group). Relative risks (RR) were adjusted for age at diagnosis of disease. Univariate analysis (Table 2) indicated that persistence of cancer (p = 0.01), delayed radioiodine ablation (p = 0.01) and pre-existing locoregional or distant metastases (p = 0.02) remained significant factors of progression of thyroid carcinoma. According to univariate analysis, the differentiated progression of thyroid cancer during pregnancy did not differ from that of non-pregnant women of the same age, unadjusted risk ratio was 1.36 (95% confidence interval from 1.14 to 1.58), but in the multivariate analysis the adjusted risk ratio was 1.04 (95% confidence from 0.91 to 1.32) (Table 2).

**Table 2:** predictive factors of the progression/ recurrence of thyroid carcinoma

	Pregnant women		Control Group		Univariate Analysis		Multivariate Analysis	
	(N = 42)	%	(N=75)	%	uRR	95% CI	aRR	95% CI
Persistence of cancer	12	28	23	30	0.62	0.44-0.84	0.52	0.32-0.68
Stage at diagnosis					0.58	0.38-0.78	0.36	0.24-0.62
T1	22	53	38	50				
T2	10	24	22	29				
T3	6	13	10	14				
T4	4	10	5	7				
N0	24	57	30	40				
N1	10	24	35	46				
Radio-iodine ablation	9	16	49	65	0.74	0.52-0.92	0.58	0.44-0.74
Months of follow-up	14.45 ± 2.4	-	18.24 ± 3.7	-	-	-	-	-
Thyroglobulin preconception (ng/ml)	1.75	-	1.88	-	-	-	-	-
Distant metastases	3	25	18	24	0.49	0.34-0.64	0.32	0.22-0.52
Pregnancy	42	100	0	0	1.36	1.14-1.58	1.04	0.91-1.32

## Discussion

Differentiated thyroid cancer is more common in women. It is the second most diagnosed cancer during pregnancy and postpartum period, with a prevalence of 14 per 100,000 live births [1,3]. Several studies have suggested an association between PTC and reproductive hormones such as estrogen and HCG [3,13]. The influence of pregnancy on the natural history of thyroid cancer is controversial. Significant effect before and during pregnancy has not been found in most studies. HCG, which is a weak agonist of the thyroid, [14,15] and various growth factors, are involved [16]. Few studies on the impact of pregnancy on thyroid cancer were conducted in thyroid cancer survivors. Leboeuf *et al*. [17] have compared the results of imaging and serum Tg in pre- and post-partum in 36 survivors of thyroid cancer. They concluded it is unlikely that pregnancy causes significant relapse in postpartum once remission has been declared.

A retrospective study by Budak *et al*. [18] was performed on 72 patients treated with thyroidectomy and radioiodine therapy; 36 of the population studied became pregnant after treatment, and 36 were non-pregnant, there were no difference between the two groups in treatment effects and prognosis (local recurrence or metastases) at follow-up. Beksaç *et al*. [5] showed, through a comparative study of a group of pregnant women followed for PTC, and a group of non-pregnant women also followed for PTC that there is no difference between the groups in terms of metastasis or relapses before and after pregnancy. Hirsch *et al*. [6] tested 63 women who gave birth after receiving treatment and compared their thyroid assessment of postoperative monitoring before and during pregnancy. Their results showed no correlation between disease progression during pregnancy and the following parameters: stage of PTC, interval between diagnosis and pregnancy, TSH level during pregnancy, and thyroglobulin before conception. They also noted a strong correlation between cancer progression and its persistence before pregnancy. This is consistent with our study, over an average follow-up duration of 2.8 years, cancer progression was correlated with the persistence of thyroid cancer before pregnancy and delay of radioiodine therapy.

The interval between diagnosis and pregnancy, the TSH levels during pregnancy or thyroglobulin level before conception had no statistically significant impact on the progression of thyroid carcinoma. Anxiety in cancer patients who wish to become pregnant is very high [19]. In our study, the average age of patients was higher than the general population. It is because most women chose to wait for remission before becoming pregnant to achieve better outcomes for mother and fetus. The lengthy period of treatment and awareness of fetal teratogenic effect of radioiodine-therapy [20] seem to force women to delay pregnancy, hence the importance of planning the pregnancy after remission. Our study showed that pregnancy did not constitute a factor on progression of thyroid carcinoma. Limitations of this study are the relatively small number of patients and the short follow up period.

## Conclusion

Pregnancy has no significant effect on the recurrence of thyroid carcinoma in patients exhibiting no persistence of the disease before conception. Only a delay in treatment, in particular a delay in radioiodine therapy, could affect the prognosis in patients not declared remitted before pregnancy. However, regular monitoring of patients with thyroid cancer in pregnancy is still important.

### What is known about this topic

- Thyroid carcinoma is more frequent in women of reproductive age;
- It can affect both fertility and the course of pregnancy.

### What this study adds

- The study established that pregnancy has no effect on the recurrence of thyroid carcinoma in patients exhibiting no persistence of the disease before conception.
